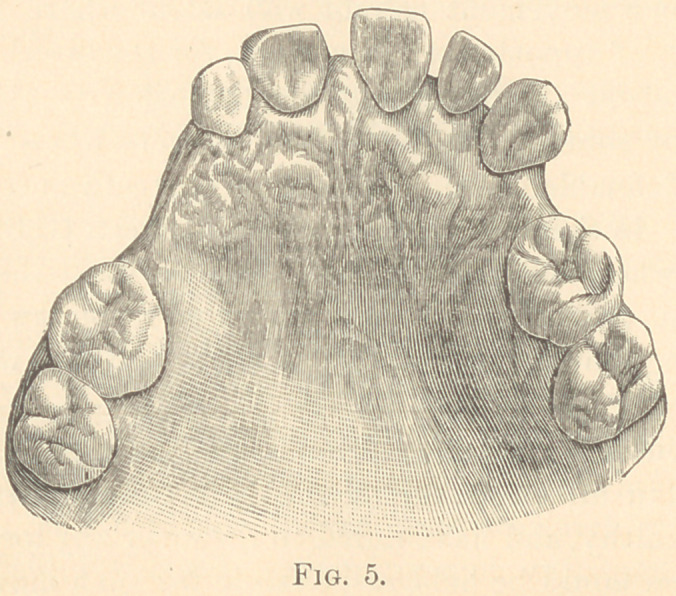# Voluntary Tooth Movement, Resulting in Excessive Inter-Dental Spaces

**Published:** 1889-03

**Authors:** S. H. Guilford

**Affiliations:** Philadelphia


### VOLUNTARY TOOTH MOVEMENT, RESULTING IN EXCESSIVE INTER-DENTAL SPACES.*

BY S. H. GUILFORD, D.D.S., PH.D., PHILADELPHIA.

Notwithstanding the full consideration that the subject of irregularity of the teeth has received at the hands of investigators and writers in recent years, there is one phase of the subject that has as yet received little or no attention.

I refer to that condition in which the teeth, instead of remaining in contact with each other as they normally should, become separated by spaces more or less great, constituting a deformity

* Read at the Tenth Anniversary Meeting of the Odontological Society of Pennsylvania, December 13, 1885.

that is not only unsightly, but one that carries with it the possibility of lessened usefulness to the individual.

All of us, at times, have seen cases in which this condition was present; but as the deformity was not a very serious one, as compared with others that we are accustomed to meet with, or, perhaps, because there seemed to be no ready way of correcting or preventing it, its real importance has been overlooked and its etiology not been inquired into.

I bring the subject to your notice on this occasion in the hope that by interchange of opinion and subsequent investigation we may be able to arrive at some conclusion in reference to its etiology, and devise means for its prevention.

The condition presents itself to us in a variety of form and degree and under varying circumstances. Not infrequently during the earlier period of second dentition the erupting teeth, especially the anterior ones in the superior arch, assume a regular position so far as the line of the arch is concerned, but they fail to approximate as they should, thus leaving spaces between them. When the later erupting teeth seek their proper position the force exerted by their pressure will usually obliterate the pre-existing spaces by causing the neighboring teeth to move into contact. Should certain teeth be lacking, however, or fail to erupt, the interdental spaces will remain as a permanent disfigurement. In some cases, as where the superior lateral incisors are lacking, these spaces are often excessive, and we have no other means of remedying the deformity than the insertion of an artificial tooth.

Fig. 1.

Fig. 1 represents a case of this kind that lately came under the writer's care.

The space between the superior centrals is nearly as great as the width of one of the adjoining teeth. Inquiry developed the fact that these centrals erupted in their present positions. The laterals were so much delayed in eruption that when they appeared the cuspids had already taken position next to the centrals. As a consequence the laterals were obliged to find room inside of the arch, and sometime later were extracted. The patient is a lady about twenty-five years of age. All of the other teeth are in normal position.

Interdental spaces of less extent are also frequently found in young patients, which are clearly due to the peculiarities of inheritance. A child inheriting the large jaw of one parent, with the small teeth of the other, cannot help but have spaces between them, from the fact that the united diameters of the teeth are not as great as the length of the arch. In such cases the spaces are usually very evenly distributed between the ten anterior teeth.

Another instance of the occurrence of interdental spaces after maturity is passed is when the six anterior teeth of the superior arch are gradually forced forward and outward until they assume a fan-like appearance, and protrude at quite an angle from the nearly vertical line of their normal position. Fig. 2 represents this condition.

Fig. 2. (After Talbot.)

Protrusion without spaces indicates either a crowded condition of the teeth, too large teeth for the jaw, or an hereditary mal-relation between the two jaws. Protrusion with spaces, however, is of an entirely different character, and indicates that the teeth have been forced out of their positions after eruption by some mechanical agency. An examination of this class of cases will reveal the fact that there is an abnormal over-bite, due, in most cases, to short molar crowns and long inferior incisors. The impact of the lower incisors and cuspids against the inclined palatine surfaces of the superior ones, causes them in the course of time to yield to the force and move outward into mal-position.

It is not, however, of the deformities just referred to that I wish to speak, for in each of these cases the causes responsible are plainly apparent and well understood.

I desire, instead, to direct your attention to that other class of cases in which, at maturity, the teeth of each jaw are found to be in normal position and contact, but where, later in life, spaces appear between the teeth of either jaw, at one or more places. The formation of these spaces is very gradual; so much so that, to attain any considerable extent, a period of five, ten or more years is often involved. For this reason they are seldom noticeable before the patient has reached the age of twenty-five or thirty years.

The slow increase of these spaces may be due in part to the character of the cause producing them; but it is probably especially due to the fact that in most cases the movement of many teeth is necessary to their formation.

In addition to the slowness of growth of these interdental spaces, there is another peculiarity associated with them, and that is the absence cf any apparent cause for their occurrence. It is this fact that has led me to study this form of tooth movement.

The condition is certainly an anomalous one, for we see these spaces occurring between the teeth of individuals of varying ages, without regard to sex, condition of health, or peculiarity of tooth or alveolar structure.

The problem that presents itself for our solution is: Why should this condition be met with, and what are the causes operative in its production?

Few theories have as yet been advanced to account for it.

Where a single space exists, it is usually found on the median line, between the superior or inferior central incisors. This fact has led some to infer and advance the theory that the space has been caused by a gradual thickening or enlargement of the alveolar septum; which would seemingly be most likely to occur in the line of union of the superior maxillary bones, or the two halves of the inferior maxilla. If such space should occur through the enlargement of the septum, the causes producing such enlargement would still remain to be explained. More than this, the space thus caused would naturally have to be filled by the enlarged septum in its full extent. Such, however, is not the case, as we can readily see by examining any case in which the space is unusually large, as in Fig. 1. In this case, it will be noticed that there is quite a depression in the alveolar outline, similar to that which occurs after a tooth has been extracted. Again: If the interstitial growth along the median line should be held responsible for median separations, it would fail to account for similar spaces occurring some distance from that line, as illustrated in Fig. 3, where the separation has occurred between the central and lateral incisors. We must look further than this for the cause.

Fig. 3.

Other practitioners have urged the theory that excessive interdental spaces are produced by the extraction of some of the posterior teeth; and that the anterior ones, being thus relieved of their support and pressure, naturally fall back somewhat towards this space. While this theory does not explain the cause of tooth movement, it certainly indicates one of the factors in such movement, and to this extent is correct; for we could have no backward movement of the teeth without space having been provided for the purpose.

All observers have noticed the frequency with which separations occur between teeth immediately anterior to the space created by the extraction of one of the larger teeth; as, for instance, between the bicuspids when the first permanent molar has been removed. Such movement cannot well occur where the occlusion with the teeth of opposite jaw is normal, for the interlocking of the cusps will prevent it; but where the occlusion is abnormal, such falling back of some of the side teeth is very likely to occur and without any definable cause. Why do these teeth move when room is provided? Is there so much pressure between teeth normally in contact that when relieved they are forced toward the space, or is the movement the result of that variation from law so often noticeable throughout nature, which has not as yet met with an explanation? I think, the latter. In the investigation of cases of this kind, where but a few teeth anterior to the space have moved backward, I have, in all cases, found the soft tissues surrounding the teeth to be healthy, and have failed to discover any local or general conditions that could be held responsible for such movement.

I therefore think that in cases of this limited character we are justified in assuming that the movement is caused by that undefinable tendency of these organs to wander from their normal positions whenever the opportunity occurs.

Fig 4.

In the larger class of cases, however, where separations do not occur in proximity to the created space, but at some distance from it, and where a number of teeth have to be moved in order that the separation may occur, we must look for some cause which shall be great enough to produce the pressure necessary for the movement of so many teeth.

Interdental spacing of the character just described we find exhibited in a variety of forms. We may have it as a single space between the central incisors of either jaw, as shown in in Fig 4, or at some other point not far distant, as shown in Fig. 3. We may see it manifested in the form of regular or irregular spaces between several adjoining teeth, resulting in such case, in the superior arch, in forcing the teeth out of line and producing among the anterior teeth the same fan-shaped condition as often results from abnormal occlusion.

Again we see it, although comparatively rarely, in the teeth on one side of the median line, while those on the opposite side are in

Fig. 5.

nowise affected. Fig. 5 represents a case of this character. The patient is a lady about 25 years of age. You will notice that large spaces exist between the centrals, between the central and lateral, and between the lateral and cuspid, while the two remaining teeth on the opposite side of the median line are in true position and contact. In the course of the formation of these spaces the three teeth have not only been pressed apart, but they have also been forced forward out of the line of the arch. Originally all of these five teeth were normally aligned and in contact, but about ten years ago the separations began to form, and increasing year by year, they recently seemed so disfiguring that she insisted upon having them extracted. In a model made from an impression taken after the extraction, I have inserted the teeth in the position they occupied before removal.

In all the cases coming under my notice, where one or more separations occurred in the midst of a number of firm and regular teeth, I have observed that the gum tissue in the interdental space was hypertrophied and inflamed. The uniform occurrence of the inflamed tissue in connection with the spaces, naturally suggested the idea that either the same underlying cause was responsible for both conditions, or that one of them was intermediate between the ultimate cause and effect.

The result of the placing of rubber or any expansible substance between teeth is well known to us all. Where teeth are in normal contact, many of them on either side of the elastic substance will have to move before the desired space is obtained. The size, firmness, or number of the teeth is no bar to their movement if time be allowed for it.

Now, the tough elastic tissue that covers the alveolar arch, or passes between the teeth, is of just such character that if irritated and consequently enlarged would produce considerable pressure upon the adjoining teeth, and such pressure would necessarily, in the course of time, cause them to move apart. If, then, teeth are capable of being moved from this cause, and it is entirely reasonable to suppose that they can be, it only remains for us to ascertain the cause of the irritation and consequent turgidity of the gum tissue.

We need not look far for this cause. We can find it in the form of calculus, either salivary or serumal, that has been formed on the root and lies concealed beneath the gum. Such accretions can be found in all, or nearly all cases where the gum tissue has become inflamed at certain points without any outside or mechanical influence.

The belief that calculus is the prime cause of the movement of teeth in the cases now under consideration is strengthened by the fact that you will always find it on the side of the tooth opposite to the direction in which it is moving. This cannot well be demonstrated on teeth in the mouth, but it is easily noticeable on those that have been extracted. In the case illustrated in Fig. 5, each of the three irregular teeth moved in an outward and backward direction, and on the roots of each of them was found a formation of calculus at a point where the mesial and lingual surfaces joined. An examination of the central tooth in model 5, which is removable, will reveal this condition.

In all of the last three cases mentioned, as well as in numbers of others of like character, an accumulation of calculus was found upon the roots of one or both teeth adjoining the separation and always upon the side opposite to the direction in which the teeth has moved.

From what has been said and from the facts presented, we are justified, I think, in arriving at the following conclusions:

1st. Where teeth have become separated in the line of the arch, opportunity for such movement has been furnished by the extraction of one or more teeth; usually the first permanent molars.

2d. Where only a few teeth have moved out of position, and they are situated next to the tooth that has been extracted, their movement is due to an unexplained tendency on the part of teeth to change their position when circumstances favor it.

3d. Where interdental spaces occur at a distance from the point where teeth have been lost, they are caused by the gradual and continuous pressure of inflamed gum tissue, and such irritation is directly due to a deposit of calculus at some point upon the roots of the separated teeth.

Having thus considered the etiology of the condition, there remains for us still to devise some means for its correction or prevention.

As to its correction after it has progressed to any considerable degree, no method has as yet been devised, and considering the usual age of the patient when the worst forms of the condition are reached, added to the fact that the teeth are then often loose, it seems extremely probable that any effort to bring them back to their former positions and have them again grow firm would be unsuccessful.

Our efforts, for the present, at least, must be directed toward the prevention or the amelioration of the unfortunate condition.

To this end, when the first manifestations appear, the root of the moving teeth should be thoroughly explored for calcareous accretions, and the same removed, if found. The gums should then be treated by applications of such astringent and alterative remedies as are likely to restore the tissues to their normal conditions. The occlusion of the opposing teeth should also be carefully noticed, and, if found necessary, they should be reduced on their cutting edges ; for undue contact may have been one of the minor causes of the condition in its earliest stages.

The best means of prevention, however, lies in the preservation of the full complement of teeth, thus avoiding, in most cases, the possibility of, as well as the inducement for lateral movement and the creation of spaces. For regulating and other purposes, certain teeth, especially the first permanent molars, are often ruthlessly sacrificed. In the effort to simplify a somewhat difficult case of regulating, teeth are often removed without a just conception of the harm that may afterward result from such action. One difficulty is gotten rid of, only to be followed somewhat later by a greater one, not so easily dealt with.

It is often best and even necessary to sacrifice one or more teeth to attain the most desirable results in regulating, but it should never be done hastily, thoughtlessly or without full consideration of the possible ill-results that may manifest themselves at some other point in the arch.

## Figures and Tables

**Fig. 1. f1:**
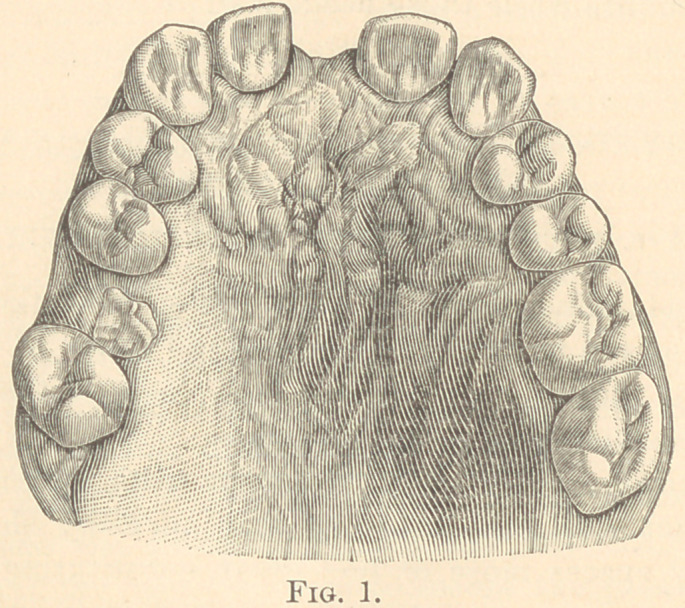


**Fig. 2. f2:**
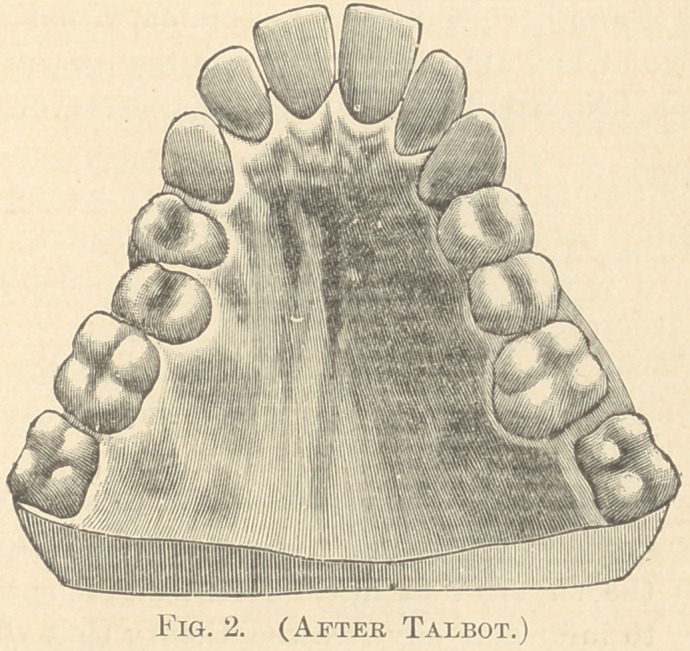


**Fig. 3. f3:**
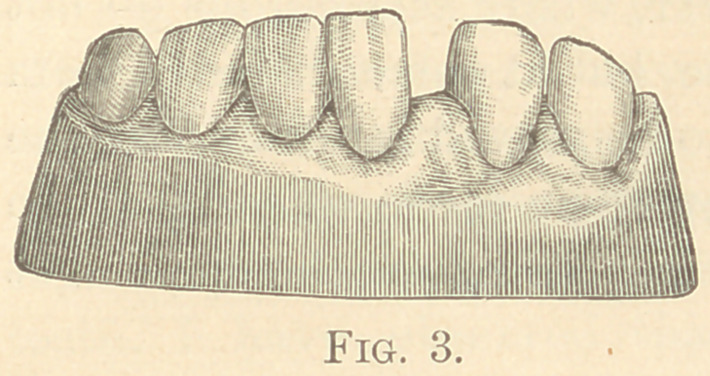


**Fig. 4. f4:**
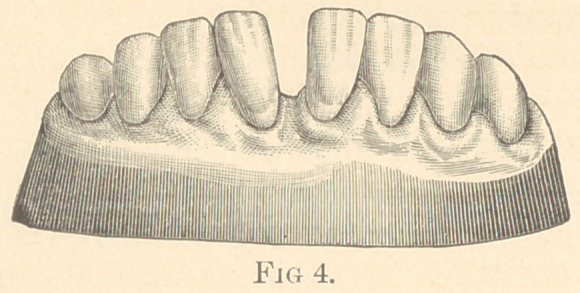


**Fig. 5. f5:**